# Organizing Pneumonia Induced by Tocilizumab in a Patient with Rheumatoid Arthritis

**DOI:** 10.7759/cureus.6982

**Published:** 2020-02-13

**Authors:** Pedro A Gouveia, Eduarda Carneiro Gomes Ferreira, Paulo M Cavalcante Neto

**Affiliations:** 1 Internal Medicine, Hospital Das Clinicas, Federal University of Pernambuco, Recife, BRA; 2 Internal Medicine, Hospital Das Clínicas, Federal University of Pernambuco, Recife, BRA; 3 Internal Medicine: Pulmonology, Institute of Medical Assistance to the State Public Servant, São Paulo, BRA

**Keywords:** rheumatoid arthritis, interstitial lung disease, organizing pneumonia, tocilizumab, drug-induced pneumonia

## Abstract

Interstitial lung disease is a significant extra-articular manifestation of rheumatoid arthritis, due to its prevalence, morbidity and mortality. Biological therapies are widely used for rheumatoid arthritis treatment. However, some biological agents have been related to the induction or exacerbation of interstitial lung disease. We report a 51-year-old woman with knee arthralgia, hand and foot joint deformities. Although there were no respiratory symptoms, rheumatoid arthritis and interstitial lung disease were diagnosed. High-resolution computed tomography (HRCT) detected a radiological pattern of nonspecific interstitial pneumonia. After tocilizumab therapy for nine months, a second HRCT detected a worsening of interstitial lung disease, presenting a pattern of organizing pneumonia. Tocilizumab was discontinued and prednisone (1 mg/kg/day) was introduced. After two months, a further HRCT detected a significant improvement in organizing pneumonia. There are few similar cases in the literature of tocilizumab-induced organizing pneumonia in patients with rheumatoid arthritis. Despite being a rare adverse effect, knowledge of this association is important for monitoring the use of tocilizumab.

## Introduction

Interstitial lung disease (ILD) is a frequent pulmonary manifestation of rheumatoid arthritis (RA) contributing to its morbidity and mortality [1]. ILD may be related to the chronic inflammatory process of RA itself, as well as to the immunomodulation of disease-modifying antirheumatic drugs (DMARDs) used in treatment [2]. Synthetic chemical compounds (csDMARDs) and biological agents (bDMARDs) have been related to the induction or exacerbation of ILD, making it difficult to choose a safe and effective therapeutic approach [2,3]. We report a RA patient who developed organizing pneumonia (OP) after treatment with tocilizumab (TCZ), an interleukin-6 (IL-6) receptor blocking monoclonal antibody.

## Case presentation

A 51-year-old woman referred to an outpatient clinic due to bilateral knee arthralgia, severe joint pain in the proximal metacarpophalangeal and interphalangeal joints with 30-minute morning stiffness. Although she had smoked until seven years ago, she denied respiratory symptoms. Her physical examination revealed wrist and metacarpophalangeal joint blocking, ulnar drift of the fingers, swan neck deformities, and fibular drift of the toes. Rales were audible in lower thirds of the lung fields, and the oxygen saturation was 97%. Laboratory tests revealed rheumatoid factor 110 U/mL (normal, <20 U/mL), anticitrullinated protein antibody 57 U (normal, <10 U), C-reactive protein 13 mg/dL (normal, < 0.5 mg/dL) and the erythrocyte sedimentation rate 97 mm/hour. RA diagnosis was performed with the Disease Activity Score (DAS28) 4.03, corresponding to a moderate disease activity.

**Figure 1:**
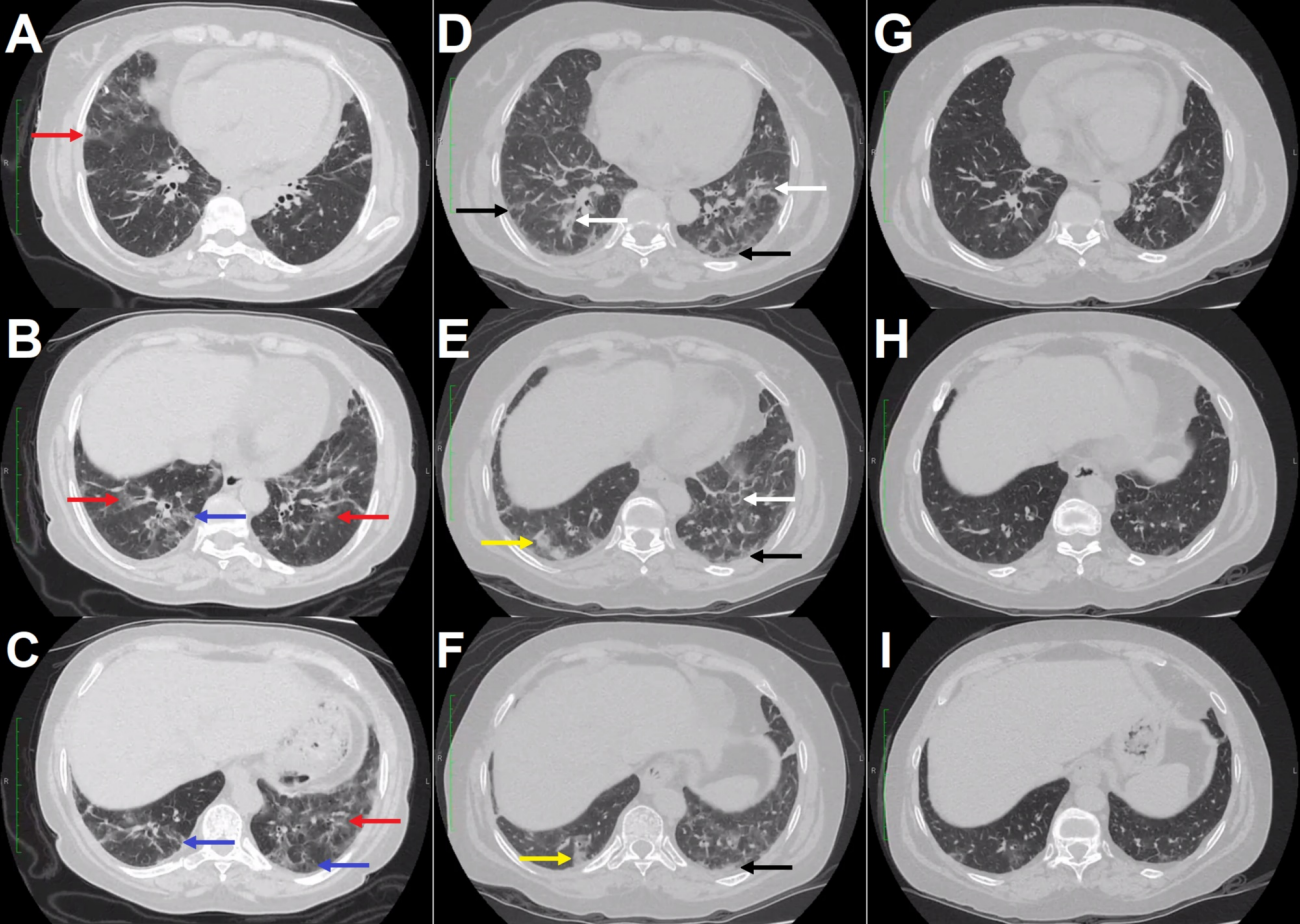
High-resolution computed tomography images of the patient with rheumatoid arthritis. Ground-glass opacities (red arrows) predominant in the lower third of the lungs, associated with mild traction bronciolectasis (blue arrows), suggesting nonspecific interstitial pneumonia (A-C). Increased areas of ground-glass opacities of a predominantly perilobular and peribronchovascular pattern (white arrows), associated with reticulation (black arrows) and bilateral patchy consolidations (yellow arrows), more evident in lower lobes, suggestive of organizing pneumonia (D-F). Significant reduction in ground-glass and consolidative opacities after tocilizumab suspension and cortiscorteroids onset (G-I).

High-resolution computed tomography (HRCT) was suggestive of nonspecific interstitial pneumonia (NSIP) (Figure 1A-C). Spirometry revealed a restrictive disorder with a forced vital capacity (FVC) of 46% of the predicted level, a forced expiratory volume in the first second (FEV1) of 48% of the predicted value and a FEV1/FVC ratio above the lower limit of normality. Bronchoscopy with bronchoalveolar lavage culture for bacteria and fungi and polymerase chain reaction for Mycobacterium tuberculosis were negative. RA-ILD diagnosis was made after excluding other causes and treatment was initiated due to the severity of the findings. We opted for a monthly dose of TCZ (8 mg/kg) and prednisone 0.5 mg/kg/day, as several csDMARDs and bDMARDs were associated with the induction or exacerbation of lung disease. The patient presented an improvement of joint symptoms and low disease activity with DAS28 of 2.8, with a gradual withdrawal of prednisone.

After nine months of continuous TCZ therapy, a chest HRCT was performed and was suggestive of OP (Figure 1D-F). A bronchoscopy with bronchoalveolar lavage culture detected no infection. New spirometry also demonstrated restrictive disorder with lower values of both FVC and FEV1 (44% and 37% predicted, respectively). In view of the significant worsening of the radiological pattern for OP, TCZ therapy was discontinued. Prednisone 1 mg/kg/day was introduced to OP treatment. After two months, a new chest HRCT was performed and presented a significant improvement in findings compared to the previous examination (Figure 1G-I). Prednisone was gradually reduced and tofacitinib initiated due to joint activity. The patient had a good response to treatment with joint and pulmonary improvement.

## Discussion

Pulmonary manifestations of RA usually occur in the first five years of disease and in some cases precede joint symptoms [4,5]. Patients with RA may have low functional capacity due to joint deformities, and therefore do not develop respiratory symptoms, such as the current case. Crackles in the lower third of the lungs on physical examination led us to investigate ILD. Initial diagnosis of RA-ILD is important, since methotrexate (MTX), a first-line drug in RA treatment, may increase a small risk for interstitial pneumonitis [2,3]. Therefore, physicians should be aware of diagnosing ILD in all patients with RA, and not be restricted to those who are symptomatic.

RA-ILD presents several histopathological and radiological patterns. The most frequent patterns are usual interstitial pneumonia in 40%-62% and NSIP in 11%-38% [3,4]. OP is a less common pattern and may reach 11% of RA-ILD [4]. There is no clear difference in clinical features or radiological findings between OP secondary to RA and cryptogenic organizing pneumonia (COP). Initial symptoms are usually dyspnea, cough and constitutional symptoms. Most common HRCT findings are bilateral involvement with random peripheral and central lesions in the middle and lower third of the lungs. The following radiological patterns are most identified in OP: ground-glass opacities (88.9%), consolidation (83.3%) and peribronchovascular opacities (52.8%) [6]. Lung biopsy is the gold standard for COP diagnosis. However, association of clinical and radiological findings typical of OP in RA patients is sufficient for diagnosis [5,7].

Pérez-Dórame et al. reported a correlation between RA activity scores and the ground-glass score present on chest HRCTs, suggesting that inflammation in RA-ILD is secondary to RA disease activity [8]. Akiyama et al. corroborated this hypothesis proving the relationship between the acute exacerbation of RA-ILD and uncontrolled arthritis activity [9]. In the present case, the patient improved from joint inflammation and presented low DAS28 activity after TCZ initiation. This response to treatment suggests ILD exacerbation was due to bDMARD rather than RA activity.

New-onset and exacerbation of ILD have been reported in association with several biological therapies [1]. Tumor necrosis factor (TNF) inhibitors stand out, affecting up to 97% of bDMARD-associated ILD cases. A relation between TCZ, an IL-6 receptor blocking monoclonal antibody, and ILD is not yet universally established in the literature. However, a systematic review conducted in 2011 identified non-infectious pulmonary adverse effects in six cases (1.0%) amongst 589 patients undergoing treatment with TCZ for RA [10].

Several case reports suggest that OP may be caused by toxicity of bDMARDs [11-13]. Mori et al. identified 21 Japanese patients with OP and RA. Twelve patients developed OP after biological onset, one with TCZ and the others with TNF inhibitors [7]. A causal relationship between drug toxicity and lung disease is reinforced because most patients had control of disease activity. Similarly, the patient reported here presented low activity in DAS28.

Two cases of TCZ-associated OP have been previously reported in RA patients. In the first case, Ikegawa et al. described a 66-year-old woman with RA who initiated TCZ after MTX therapy for two years. Three days after the first TCZ infusion, she was diagnosed with OP and elevated transaminases, which improved after discontinuing TCZ and initiating corticosteroid [12]. In the second case, Mori et al. identified a 63-year-old man with RA activity controlled by TCZ therapy for 25 months, in combination with MTX and tacrolimus. OP was diagnosed and TCZ was withdrawn with prednisolone 30 mg/day inducing a recovery from ILD [7]. The current case is the third to describe TCZ-associated OP in RA patients; however, we present an exacerbation of a previous ILD. Use of the Naranjo adverse drug reaction probability scale indicates the strength of the causal relationship as probable in the present case [14].

All three TCZ-associated OP cases presented excellent responses by discontinuing TCZ and introducing corticosteroid therapy [7,12]. OP is known to respond better to corticosteroids than other RA-ILD subtypes [1]. RA reactivation is a concern after TCZ has been discontinued. Reintroducing biological therapy is difficult, and it is therefore more prudent to consider using another bDMARD. After the 12 cases of improved OP associated with bDMARD, Mori et al. maintained three patients with low disease activity without medication and introduced another bDMARD or MTX in the remaining cases [7]. Although MTX may increase a small risk for interstitial pneumonitis, Kiely et al. recently identified no association between MTX exposure and RA-ILD incidence in two cohorts (ERAS and ERAN) [2,3,15]. We chose to introduce tofacitinib because there is no association described with ILD.

## Conclusions

We report a TCZ-associated OP case in a patient with RA, with few similar cases described in the literature. Even with pulmonary severity, interruption of TCZ and corticosteroid initiation were enough to resolve this case. For better elucidation of TCZ lung safety, clinical trials comparing efficacy and safety of different bDMARDs in this clinical setting are required. Despite being a rare adverse effect, knowledge of this association is important for monitoring the use of TCZ.
